# Catalytically inactive Cas9 impairs DNA replication fork progression to induce focal genomic instability

**DOI:** 10.1093/nar/gkaa1241

**Published:** 2021-01-04

**Authors:** Goro Doi, Satoshi Okada, Takehiro Yasukawa, Yuki Sugiyama, Siqin Bala, Shintaro Miyazaki, Dongchon Kang, Takashi Ito

**Affiliations:** Department of Biochemistry, Kyushu University Graduate School of Medical Sciences, 3-1-1 Maidashi, Higashi-ku, Fukuoka 812-8582, Japan; Department of Biochemistry, Kyushu University Graduate School of Medical Sciences, 3-1-1 Maidashi, Higashi-ku, Fukuoka 812-8582, Japan; Department of Clinical Chemistry and Laboratory Medicine, Kyushu University Graduate School of Medical Sciences, 3-1-1 Maidashi, Higashi-ku, Fukuoka 812-8582, Japan; Department of Biochemistry, Kyushu University Graduate School of Medical Sciences, 3-1-1 Maidashi, Higashi-ku, Fukuoka 812-8582, Japan; Department of Biochemistry, Kyushu University Graduate School of Medical Sciences, 3-1-1 Maidashi, Higashi-ku, Fukuoka 812-8582, Japan; Kyushu University School of Medicine, 3-1-1 Maidashi, Higashi-ku, Fukuoka 812-8582, Japan; Department of Clinical Chemistry and Laboratory Medicine, Kyushu University Graduate School of Medical Sciences, 3-1-1 Maidashi, Higashi-ku, Fukuoka 812-8582, Japan; Department of Biochemistry, Kyushu University Graduate School of Medical Sciences, 3-1-1 Maidashi, Higashi-ku, Fukuoka 812-8582, Japan

## Abstract

Catalytically inactive Cas9 (dCas9) has become an increasingly popular tool for targeted gene activation/inactivation, live-cell imaging, and base editing. While dCas9 was reported to induce base substitutions and indels, it has not been associated with structural variations. Here, we show that dCas9 impedes replication fork progression to destabilize tandem repeats in budding yeast. When targeted to the *CUP1* array comprising ∼16 repeat units, dCas9 induced its contraction in most cells, especially in the presence of nicotinamide. Replication intermediate analysis demonstrated replication fork stalling in the vicinity of dCas9-bound sites. Genetic analysis indicated that while destabilization is counteracted by the replisome progression complex components Ctf4 and Mrc1 and the accessory helicase Rrm3, it involves single-strand annealing by the recombination proteins Rad52 and Rad59. Although dCas9-mediated replication fork stalling is a potential risk in conventional applications, it may serve as a novel tool for both mechanistic studies and manipulation of genomic instability.

## INTRODUCTION

Cas9 is an RNA-guided endonuclease that cleaves double-stranded DNA at its target site, and the ease of designing single-guide RNA (sgRNA) has made it the most popular tool in genome editing. Catalytically inactive Cas9 (dCas9) bears mutations at two nuclease domains and has enabled a variety of applications. For instance, dCas9 can inhibit the progression of RNA polymerase to suppress transcription of the gene to which it binds (CRISPRi) (1). Moreover, dCas9 has been fused or complexed with fluorescent proteins, transcriptional activation/repression or epigenetic modification domains, and adenosine/cytidine deaminases to enable live-cell imaging of genomic loci, targeted gene activation/inactivation, and base editing, respectively (2). These applications take advantage of the function of dCas9 as a programmable sequence-specific DNA-binding protein. Since dCas9 lacks nuclease activity, it was presumed to be non-mutagenic. However, it was also reported to promote mutagenesis at a frequency of ∼10^−5^ via R-loop formation (3). Most of these mutations were base substitutions attributable to spontaneous cytosine deamination of the non-target DNA strand of the dCas9-induced R-loop, whereas others included homopolymer instability and trans-lesion synthesis (TLS) (3). While dCas9 induces base substitutions and small indels, it has not been demonstrated, to our knowledge, to induce large structural variations (SVs). As recent studies reported Cas9 often induces unexpectedly large deletions around its target sites (4,5), the impact of dCas9-binding to genome DNA in vivo should be carefully examined in terms of SVs.

Eukaryotic genomes harbor many repetitive sequences in the form of tandem or interspersed repeats (6). They occasionally induce genomic instability leading to generation of SVs, including both pathogenic and adaptive copy number variations (CNVs). The most famous disease related to CNV of tandem repeats is triplet repeat disease, which is evoked by the expansion of arrays comprised of very short units, such as CAG, GAA, CGG, and CCG trinucleotides (7). In contrast, facioscapulohumeral muscular dystrophy is caused by the contraction of D4Z4 macrosatellite repeats comprised of a 3.3-kb unit harboring the *DUX4* gene (8). CNV-mediated environmental adaptation has been well documented in budding yeast (9). When exposed to high concentrations of copper ions, yeast cells amplify the resistance gene *CUP1* to rapidly generate adapted progenies. Most yeast strains contain tandemly iterated copies of *CUP1* gene. Intriguingly, the presence of copper was shown to accelerate intra/sister chromatid recombination rate at *CUP1* array (10). This enhanced recombination likely contributes to the generation of expanded *CUP1* arrays and, hence, adapted progenies with enhanced copper resistance. Another example is rDNA comprising ∼150 copies of tandemly iterated units, and its instability is involved in cellular senescence (11). Both examples notably involve replication fork stalling or collapse followed by its repair.

The replisome at the replication fork uses the Cdc45–Mcm2–7–GINS complex (CMG helicase) to unwind DNA for fork progression and DNA synthesis by replicative polymerases. The replicative CMG helicase associates with proteins in the replisome progression complex (RPC), which includes the checkpoint mediator Mrc1, the Tof1–Csm3 complex, the replisome adaptor protein Ctf4, the histone chaperone FACT and DNA topoisomerase I (12). Replication fork stalling occurs through the actions of these proteins upon encountering an obstacle. The accessory helicase Rrm3 removes DNA-bound proteins such as the origin recognition complex, the transcription regulator Rap1, and the replication fork-blocking protein Fob1, whereas the Tof1–Csm3 complex counteracts Rrm3 in the removal process (13,14). Depletion of these proteins induces the contraction or expansion of tandem repeats. For instance, *ctf4*Δ cells amplify the copy number of rDNA (15), and *mrc1*Δ or *rrm3*Δ cells destabilize both the *CUP1* and rDNA arrays (14,16–18). Prolonged replication fork stalling results in its collapse, with or without DNA double strand breaks (DSBs). Cells have several pathways for coping with collapsed/broken forks, including homologous recombination (HR), non-homologous end joining (NHEJ), break-induced replication (BIR), TLS, template switching (TS) and single-strand annealing (SSA). During the repair process, repetitive sequences around the collapsed fork occasionally trigger the generation of SVs, including CNV of tandem repeat units. For the copper-accelerated *CUP1* recombination described above, a model was proposed in which activated promoter activity-induced replication fork collapse is followed by BIR or fork restart using a homologous sequence on the sister chromatid (16).

Here, we report dCas9-induced CNV of tandem repeats. This finding led us to uncover that dCas9 impairs replication fork progression to induce focal genomic instability.

## MATERIALS AND METHODS

### Yeast strains

All yeast strains used in this study are derived from BY4741 (*MAT***a***his3*Δ*1 leu2*Δ*0 met15*Δ*0 ura3*Δ*0*) (19) (Supplementary Table S1). Standard culture media and genetic methods were used in this study (20). We deleted a gene of interest by transforming yeast cells with a DNA fragment composed of KanMX cassette sandwiched by the 5′- and 3′-flanking sequences of the open reading frame of the gene, which was amplified from the corresponding deletant strain in Yeast Deletion Clones *MAT***a** Complete Set (invitrogen) using PCR primers listed in Supplementary Table S2. To construct a strain bearing a *URA3* insertion at the boundary of two neighboring *CUP1* repeat units, we transformed yeast cells with a DNA fragment composed of *URA3* cassette sandwiched by the 3′- and 5′-end sequences of the repeat unit, which was obtained by PCR with primers VIII214253::URA3-F and VIII214253::URA3-R listed in Supplementary Table S2. Transformants selected on agar plates of synthetic complete medium lacking uracil (SC−Ura) supplemented with 2% glucose were used for nanopore sequencing to determine the integration site of *URA3* in the *CUP1* array.

### Plasmids

All plasmids used in this study are listed in Supplementary Table S3. All primers for plasmid construction were purchased from Sigma-Aldrich and Eurofins Genomics. Plasmids were constructed with the seamless cloning with HiFi DNA Assembly or Golden Gate Assembly obtained from New England Biolabs (NEB).

The integrative plasmid YIplac128-pCSE4-dCas9-tADH1 (*LEU2*) harbors a gene encoding *Streptococcus pyogenes* dCas9 fused with SV40 nuclear localization signal (NLS) as described previously (21) under the control of *CSE4* promoter. It was used for yeast transformation after NruI digestion to be integrated to the *CSE4* promoter on the genome.

The integrative plasmid YIplac128-pGAL1-dCas9-tADH1 (*LEU2*) harbors a gene encoding *S. pyogenes* dCas9 fused with SV40 NLS under the control of *GAL1* promoter. It was used for yeast transformation after AgeI digestion to be integrated to the *GAL1* promoter on the genome.

The integrative plasmid pFA6a-pACT1-yGEV-tADH1-HphMX (*Hyg^R^*) harbors a gene encoding β-estradiol-responsive artificial transcription activator GEV (22) under the control of *ACT1* promoter. It was used for yeast transformation after CpsCI digestion to be integrated to the *ACT1* promoter on the genome. The GEV-coding sequence was codon-optimized for *Saccharomyces cerevisiae*.

The integrative plasmid pFA6a-pCUP2-yGEV-tADH1-HphMX (*Hyg^R^*) harbors the codon-optimized GEV-coding gene under the control of *CUP2* promoter. It was used for yeast transformation after MfeI digestion to be integrated to the *CUP2* promoter on the genome.

Centromeric plasmids for sgRNA expression harbor sgRNA gene under the control of *SNR52* promoter or *GAL1* promoter. The sgRNA scaffold sequence contains a base-flip and an extension of a stem–loop for stable sgRNA expression (23). To cut off an unnecessary sequence from the 5′-terminal portion of the sgRNA-containing transcript, each sgRNA sequence is preceded by a hammerhead ribozyme (Supplementary Table S3). To define the 3′-terminus, each sgRNA sequence is followed by *SUP4* terminator on the *SNR52* promoter plasmid or by the HDV ribozyme on the *GAL1* promoter plasmid (Supplementary Table S3). For designing sgRNAs, CRISPRdirect (24) was used to select target sites in the yeast genome listed in Supplementary Table S4.

### Gene editing

For constructing *rad52*Δ *rad59*Δ strains, we performed enAsCas12a-based gene editing. All gene-editing plasmids used in this study are listed in Supplementary Table S3. Each gene-editing centromeric plasmid (*URA3*, *CEN*) harbors a gene encoding enAsCas12a (25) fused with SV40 NLS and a gene coding CRISPR RNA (crRNA), both of which are under the control of *GAL1* promoter. To improve the efficiency of gene editing, a 9-mer sequence (U_4_AU_4_) was attached to the 3′-end of crRNA (26). The crRNA is flanked by a hammerhead ribozyme and the HDV ribozyme at its 5′- and 3′-termini, respectively. For designing crRNAs, CRISPOR (27) was used to select target sites listed in Supplementary Table S4.

Yeast cells transformed with the gene-editing plasmid were spread on agar plates of SC−Ura supplemented with 2% galactose. After incubation at 30°C for 4–5 days, colonies were picked and streaked on a new plate. To examine successful gene-editing at the target site on the genome, we performed PCR to amplify a region spanning the target site. The PCR products were sequenced to reveal the size and position of indels around the target site (Supplementary Table S1).

To eliminate the gene-editing plasmid, the successfully gene-edited strains were grown in yeast extract/peptone/dextrose (YPD) liquid medium at 30°C overnight and streaked on a YPD agar plate to isolate single colonies. After incubation at 30°C for 2–3 days, each colony was streaked on agar plates of YPD medium and SC−Ura medium supplemented with 2% glucose to confirm the loss of the gene-editing plasmid.

### Cell growth rate measurement

Cell growth rate was measured using the RTS-1 personal bioreactor (Biosan, Riga, Latvia). The properties were set as follow: the volume, 10 ml of SC medium supplemented with 2% glucose; the temperature, 25°C; the rotation speed, 1500 rpm; the measurement frequency, 10 times/min; and the reverse spin longitude, 1 s.

### Cell culture for quantitative PCR (qPCR)

Yeast cells were grown at 25°C overnight in 5 ml of SC−Ura or SC−His medium supplemented with 2% glucose. On the following day, the OD_600_ of each sample was recorded and 10–50 μl of the culture was inoculated into 5 ml of the fresh medium containing 10 nM β-estradiol, supplemented with or without 5 mM nicotinamide (NAM). From the remaining overnight culture, genomic DNA was extracted with Gentra Puregene Yeast/Bact. Kit (QIAGEN) for qPCR. The same process was repeated every day. The division number per day was calculated from the change of OD_600_.

### qPCR

The concentration of genomic DNA was measured with Qubit dsDNA BR assay on Qubit 2.0 Fluorometer or Qubit Flex Fluorometer (Thermo Fisher Scientific). The DNA solution was diluted to a concentration of 0.5 ng/μl prior to qPCR. Each qPCR solution (20 μl) contained 2 μl of DNA (1 ng), 10 μl of TB Green Premix *Ex Taq* II (Tli RNaseH Plus) (Takara), 0.4 μl of ROX Reference Dye, 2 pmol each of the forward and reverse primers. The primers used for qPCR are listed in Supplementary Table S2. Each qPCR assay was performed in duplicate, using StepOnePlus or QuantStudio3 (Applied Biosystems) according to the manufacturer's instructions. Amplification condition was initial denaturation at 95°C for 30 s followed by 40 times iteration of a 3-step thermal cycle composed of 95°C for 10 s, 55°C for 30 s and 72°C for 5 s. All qPCR runs included 10-fold serial dilutions to generate standard curves. The quantity of *CUP1*, *ENA1* and *URA3* was normalized to that of *ACT1*. The copy number of *CUP1* and *ENA1* in the standard curves was calibrated by the results of nanopore sequencing.

### Genetic assay for the loss of *URA3* inserted into the *CUP1* array

Yeast cells were grown at 25°C overnight in 5 ml of SC−His medium containing 2% glucose. On the following day, 15 μl of the culture was inoculated into 5 ml of fresh SC−His medium containing 2% glucose and 10 nM β-estradiol, supplemented with or without 5 mM NAM. The same process was repeated every day. After four days of cultivation, cells were appropriately diluted and spread onto SC glucose plates supplemented with 0.1% 5-FOA and YPAD plates to determine the frequency of 5-FOA-resistant clones.

### Nanopore sequencing

Genomic DNA was extracted using Gentra Puregene Yeast/Bact. Kit (QIAGEN) and purified with 0.4× AMPure XP (Beckman Coulter) or Short Read Eliminator kit XL (Circulomics). To obtain high molecular weight DNA, we avoided vortexing and used mixing by gentle pipetting instead. DNA libraries were prepared using the ligation sequencing kit SQK-LSK109 (Oxford Nanopore Technologies) with or without barcoding. For barcoding, we used the native barcoding kit EXP-NBD104 or the rapid barcoding sequencing kit SQK-RBK004 (Oxford Nanopore Technologies) according to the manufacturer's instructions. We modified the protocol of the ligation sequencing kit as follows: DNA fragmentation, omitted; duration of the enzymatic repair steps at 20 and 65°C, both extended from 5 min to 30 min; and the duration of ligation step, extended from 10 to 30 min. The library was sequenced with the flowcell FLO-MIN106D R9.4.1 using the MinION sequencer (Oxford Nanopore Technologies). MinKNOW software was used to control the MinION device. The run time was set to 72 h. Base calling was performed using Albacore v2.3.1, Guppy v3.6.0, or Guppy v4.0.14. The assessment of sequencing data was performed using NanoPlot (28).

### Dot plot analysis of nanopore sequencing reads

We used nanopore sequencing data in FASTA format to draw dot plots using YASS (29). We first selected reads spanning the entire array using 1-kb upstream and 1-kb downstream sequences of the *CUP1* or *ENA1* array as queries of minialign (https://github.com/ocxtal/minialign) and then used these reads as the first input sequence for YASS. As the second input, we used the reference sequence of interest (*CUP1* repeat unit, *ENA1* repeat unit, or *URA3*) or the selected read itself. By manually counting the number of diagonal lines appeared in each dot plot, we determined the *CUP1* copy number, the *ENA1* copy number and the location of *URA3* insertion in the *CUP1* array.

### Computational counting of tandem repeat units in nanopore sequencing reads

To computationally count the copy number of tandem repeat units directly from each nanopore read, we developed a Fourier transform-based program termed DNA Sequence Detector, which works as follows.

Seq1 is a long DNA sequence sample to be examined (i.e. nanopore read), whereas Seq2 is a short and known DNA sequence (i.e. reference sequence of interest).

The nucleotide sequence of Seq1 is ${s_0}{s_1} \ldots {s_{n - 1}}$, and the nucleotide sequence of Seq2 is ${r_0}{r_1} \ldots {r_{m - 1}}$. Note that ${s_i}$ and ${r_i}$ are either A, T, G or C.

Then a matrix M is created as follows:

$$\begin{eqnarray*} {\rm{M }} &=& \left[ {\begin{array}{@{}*{10}{c}@{}} {{r_0}}& \quad {}& \quad {}& \quad {}& \quad {}& \quad {}& \quad {}& \quad {}& \quad {}& \quad {{r_1}}\\ {{r_1}}& \quad {{r_0}}& \quad {}& \quad {}& \quad {}& \quad {}& \quad {}& \quad {}& \quad {}& \quad \vdots \\ \vdots & \quad {{r_1}}& \quad {{r_0}}& \quad {}& \quad {}& \quad {}& \quad {}& \quad {}& \quad {}& \quad {{r_{m - 1}}}\\ {{r_{m - 1}}}& \quad \vdots & \quad {{r_1}}& \quad {}& \quad {}& \quad {}& \quad {}& \quad {}& \quad {}& \quad {}\\ {}& \quad {{r_{m - 1}}}& \quad \vdots & \quad {}& \quad {}& \quad {}& \quad {}& \quad {}& \quad {}& \quad {}\\ {}& \quad {}& \quad {{r_{m - 1}}}& \quad {}& \quad {}& \quad {}& \quad {}& \quad {}& \quad {}& \quad {}\\ {}& \quad {}& \quad {}& \quad {}& \quad {}& \quad {}& \quad {}& \quad {}& \quad {}& \quad {}\\ {}& \quad {}& \quad {}& \quad {}& \quad {}& \quad {}& \quad {}& \quad {}& \quad {}& \quad {}\\ {}& \quad {}& \quad {}& \quad {}& \quad {}& \quad {}& \quad {}& \quad {}& \quad {}& \quad {}\\ {}& \quad {}& \quad {}& \quad {}& \quad {}& \quad {}& \quad {}& \quad {}& \quad {}& \quad {{r_0}} \end{array}} \right] \nonumber \\ &=& \left[ {{a_{i,j}}} \right]\ ,\ \left( {0 \le i,j \le n - 1} \right) \end{eqnarray*}$$

If ${s_i}$ and ${a_{i,j}}$ are the same nucleotide, ${a_{i,j}}$ is replaced with ‘1’. If ${s_i}$ and ${a_{i,j}}$ are not the same nucleotide, ${a_{i,j}}$ is replaced with ‘0’. Let the resulting matrix be M2.

$$\begin{equation*}{\rm{M}}2{\rm{\ }} = \left[ {\begin{array}{@{}*{10}{c}@{}} {}& \quad {}& \quad 0& \quad {}& \quad {}& \quad {}& \quad {}& \quad {}& \quad {}& \quad {}\\ {}& \quad {}& \quad 1& \quad {}& \quad {}& \quad {}& \quad {}& \quad 0& \quad {}& \quad {}\\ {}& \quad {}& \quad 0& \quad {}& \quad {}& \quad {}& \quad {}& \quad 1& \quad {}& \quad {}\\ {}& \quad {}& \quad {}& \quad {}& \quad 0& \quad {}& \quad {}& \quad 1& \quad {}& \quad {}\\ {}& \quad {}& \quad {}& \quad {}& \quad 1& \quad {}& \quad {}& \quad \vdots & \quad {}& \quad {}\\ {}& \quad {}& \quad {}& \quad {}& \quad 1& \quad {}& \quad {}& \quad 1& \quad {}& \quad {}\\ {}& \quad {}& \quad {}& \quad {}& \quad 1& \quad {}& \quad {}& \quad 0& \quad {}& \quad {}\\ {}& \quad {}& \quad {}& \quad {}& \quad 0& \quad {}& \quad {}& \quad {}& \quad {}& \quad {} \end{array}} \right]\ \end{equation*}$$

Next, each column in M2 is scanned. If ‘1’ appears *k* times consecutively, these ‘1’s are replaced with ‘*k*’. Let the resulting matrix be M3.

$$\begin{equation*}{\rm{M}}3{\rm{\ }} = \left[ {\begin{array}{@{}*{10}{c}@{}} {}& \quad {}& \quad 0& \quad {}& \quad {}& \quad {}& \quad {}& \quad {}& \quad {}& \quad {}\\ {}& \quad {}& \quad 1& \quad {}& \quad {}& \quad {}& \quad {}& \quad 0& \quad {}& \quad {}\\ {}& \quad {}& \quad 0& \quad {}& \quad {}& \quad {}& \quad {}& \quad {12}& \quad {}& \quad {}\\ {}& \quad {}& \quad {}& \quad {}& \quad 0& \quad {}& \quad {}& \quad {12}& \quad {}& \quad {}\\ {}& \quad {}& \quad {}& \quad {}& \quad 3& \quad {}& \quad {}& \quad \vdots & \quad {}& \quad {}\\ {}& \quad {}& \quad {}& \quad {}& \quad 3& \quad {}& \quad {}& \quad {12}& \quad {}& \quad {}\\ {}& \quad {}& \quad {}& \quad {}& \quad 3& \quad {}& \quad {}& \quad 0& \quad {}& \quad {}\\ {}& \quad {}& \quad {}& \quad {}& \quad 0& \quad {}& \quad {}& \quad {}& \quad {}& \quad {} \end{array}} \right]\ \end{equation*}$$

Next, all numbers below *X* (where *X* is a natural constant) are replaced with ‘0’. Let the resulting matrix be M4.

$$\begin{eqnarray*} {\rm{M}}4{\rm{ }} &=& \left[ {\begin{array}{@{}*{10}{c}@{}} {}& \quad {}& \quad {}& \quad {}& \quad {}& \quad {}& \quad {}& \quad {}& \quad {}& \quad {}\\ {}& \quad {}& \quad 0& \quad {}& \quad {}& \quad {}& \quad {}& \quad {}& \quad {}& \quad {}\\ {}& \quad {}& \quad 0& \quad {}& \quad {}& \quad {}& \quad {}& \quad 0& \quad {}& \quad {}\\ {}& \quad {}& \quad 0& \quad {}& \quad {}& \quad {}& \quad {}& \quad {12}& \quad {}& \quad {}\\ {}& \quad {}& \quad {}& \quad {}& \quad 0& \quad {}& \quad {}& \quad {12}& \quad {}& \quad {}\\ {}& \quad {}& \quad {}& \quad {}& \quad 0& \quad {}& \quad {}& \quad \vdots & \quad {}& \quad {}\\ {}& \quad {}& \quad {}& \quad {}& \quad 0& \quad {}& \quad {}& \quad {12}& \quad {}& \quad {}\\ {}& \quad {}& \quad {}& \quad {}& \quad 0& \quad {}& \quad {}& \quad 0& \quad {}& \quad {}\\ {}& \quad {}& \quad {}& \quad {}& \quad 0& \quad {}& \quad {}& \quad {}& \quad {}& \quad {}\\ {}& \quad {}& \quad {}& \quad {}& \quad {}& \quad {}& \quad {}& \quad {}& \quad {}& \quad {} \end{array}} \right] \nonumber \\ &=& \left[ {{b_{i,j}}} \right]\ ,\ \left( {0 \le i,j \le n - 1} \right) \end{eqnarray*}$$

${c_i}$ is defined as follows:

$$\begin{equation*}\ {c_i} = \mathop \sum \limits_{j = 0}^{n - 1} {b_{i,j}},\ (\ 0 \le i \le n - 1)\end{equation*}$$

Vector $\overset{\rightharpoonup}{v}$ is defined as follows:

$$\begin{equation*}\ \overset{\rightharpoonup}{v} = \left[ {\begin{array}{*{20}{c}} {{c_0}}& \quad {{c_1}}& \quad \ldots & \quad {{c_{n - 1}}} \end{array}} \right]\ \end{equation*}$$

Next, the program searches a region in which values are dense and larger than a certain degree. Suppose that a region *R*$ = {\rm{\ }}[ {{\rm{i}}\sim {\rm{j}}} ]$ is found as such a region. This *R* is the region in which Seq2 is located.

Next, the program finds out the number of Seq2 present in *R*. The vector $\overset{\rightharpoonup}{v}$ is smoothened to generate a vector ${\overset{\rightharpoonup}{v}_{ave}} = [ d_0\ d_1\ \ldots \ d_{n - 1}] $. ${d_i}$ is defined as follows:

$$\begin{equation*}\ {d_i} = \frac{{\left( {\mathop \sum \nolimits_{j = i - L}^{i + L} {c_j}} \right)}}{{2{\rm{L}} + 1}}\ ,\ \left( {L \le i \le n - 1 - L} \right)\end{equation*}$$

$\ {d_i} = {c_i}\ ,\ ( {i< {L,\ i} > n - 1 - L} )$, *L* is a natural constant.

By performing a discrete Fourier transform on a region *R* in ${\overset{\rightharpoonup}{v}_{ave}}$, the number of Seq2 in *R* is obtained.

### Two-dimensional agarose gel electrophoresis (2D-AGE)

Yeast cells were grown at 25°C overnight in 5 ml of SC−Ura medium containing 2% glucose. Following the addition of 10 nM β-estradiol, the cells were cultivated for 2 h, diluted, and cultivated for 4 h. The genomic DNA was extracted with Gentra Puregene Yeast/Bact. Kit (QIAGEN) using a modified protocol, in which all vortexing steps were replaced by mixing with gentle pipetting to maintain the integrity of replication intermediates. The 2D-AGE followed by Southern blot hybridization was conducted as described previously (30,31) with some modifications. In brief, two micrograms of genomic DNA was fully digested with KpnI (Takara) or XcmI (NEB), precipitated with 1/10 volume of 3 M sodium acetate and one volume of isopropanol, washed with 70% ethanol, air dried, and finally dissolved in 30 μl of 10 mM HEPES–NaOH (pH 7.2). The first-dimension electrophoresis was performed on a 0.55% agarose gel (11 × 14 cm) for 16 h at 22 V at room temperature. The second-dimension electrophoresis was performed on a 1.55% agarose gel (20 × 25 cm) containing ethidium bromide for 4 h at 260 mA at 4°C. The gel was sequentially soaked in depurination buffer, denaturing buffer, and neutralizing buffer, and the DNA was blotted to Hybond N+ membrane (Cytiva). Following UV-crosslinking, the blot was hybridized with a *CUP1* probe at 55°C overnight. The probe was generated by PCR using the primers listed in Supplementary Table S4 followed by labeling with alkaline phosphatase using the labeling module of AlkPhos Direct Labelling and Detection System kit (Cytiva). Following appropriate washing of the blot at 60°C, chemiluminescent signals were generated using the CDP-Star Detection Reagent in the kit and detected with ImageQuant LAS4000 (Cytiva). Gel images were processed with ImageJ software (National Institutes of Health) for presentation images. The process involved rotating, cropping, and altering window-level settings. The spot of interest was selected as a circle for quantification, and the total internal intensity was divided by its area. The background was defined as the mean of area-normalized intensities of three randomly selected regions with no obvious signals.

### Western blot

The expression of FLAG-tagged Rad52 was analyzed by western blotting. Proteins were extracted as described previously (32). Twenty micrograms of proteins (2 μg/μl) were separated with sodium dodecyl sulfate-polyacrylamide gel electrophoresis using Any kD Mini-PROTEAN TGX Precast Gel (Bio-Rad). Transfer to membrane was performed with iBind Western System (Thermo Fisher Scientific) according to the manufacturer's protocol. Primary and secondary antibodies to detect Rad52-FLAG were FLAG M2 mouse monoclonal antibody (1:1000, Sigma-Aldrich) and goat anti-mouse IgG-HRP (1:2000, Santa Cruz Biotechnology), respectively. Primary and secondary antibodies to detect α-tubulin (loading control) were anti-alpha Tubulin antibody [YOL1/34] (1:2000, GeneTex) and goat Anti-Rat IgG H&L (HRP) (1:2000, Abcam), respectively. Following incubation with Clarity Western ECL Substrate (Bio-Rad), chemiluminescent signals were detected with ChemiDocTouch system (Bio-Rad). Gel images were processed with ImageJ software. The process involved cropping and altering window-level settings.

## RESULTS

### dCas9 induces copy number reduction of tandem repeat units

Cup1 is a metallothionein that buffers the concentration of intracellular copper in the budding yeast *Saccharomyces cerevisiae* (33,34). A ∼2.0-kb unit including the *CUP1* gene (*CUP1* repeat unit) is tandemly iterated more than 10 times in the reference strain S288c to compose the *CUP1* array on chromosome VIII (35). The *CUP1* array in the parental strain used in this study was composed of ∼16 repeat units (see below). The level of copper resistance linearly correlates with the *CUP1* copy number (36–38). During experiments to target dCas9 to *CUP1*, we observed that the *CUP1* copy number was decreased in a strain constitutively expressing *CUP1*-targeted dCas9 (Supplementary Figure S1A). The copy number was maintained in a control strain constitutively expressing dCas9 targeted to *TEF1* on chromosome XVI (39) (Supplementary Figure S1A). Moreover, the former strain, but not the latter, showed a sign of further decrease in the copy number during cultivation (Supplementary Figure S1B).

**Figure 1.**
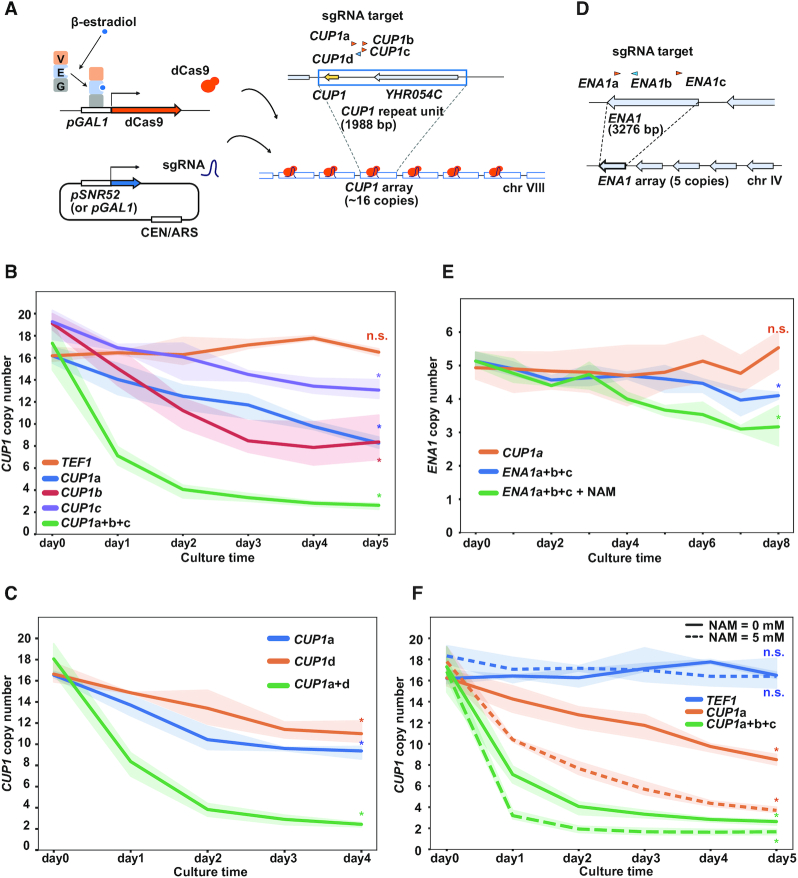
dCas9-induced copy number reduction of tandem repeat units. (**A**) Schematic of the inducible dCas9 system. GEV is composed of Gal4 DNA-binding domain (G), estrogen receptor (E) and VP16 transcription activation domain (V). GEV complexed with β-estradiol activates *GAL1* promoter (*pGAL1*) to induce dCas9. sgRNAs are expressed from a centromeric plasmid under the control of *SNR52* promoter (*pSNR52*) or *pGAL1*. Arrowheads above *CUP1* repeat unit indicate four sgRNAs (orange, Watson strand; blue, Crick strand). (**B**) Time course of *CUP1* copy number. The population-averaged copy number was determined with qPCR. Indicated sgRNAs were expressed using *pSNR52*. Expression of dCas9 was induced by the addition of 10 nM β-estradiol at day 0. Data are represented as mean ± standard deviation (i.e. line plots and shadows) (*n* = 3 or more biological replicates). Statistical significance of copy number alteration was examined between day 0 and day 5 using *t*-test (*P< 0.05). (**C**) Time course of *CUP1* copy number. Similar to Figure 1B, except that sgRNAs were expressed using *pGAL1* (*n* = 3 biological replicates). Statistical significance of copy number alteration was examined between day 0 and day 4 using *t*-test (*P< 0.05). (**D**) Schematic of *ENA1* array and sgRNAs. (**E**) Time course of *ENA1* paralog copy number. Similar to Figure 1B (*n* = 3 biological replicates). Statistical significance of copy number alteration was examined between day 0 and day 8 using *t*-test (*P< 0.05). (**F**) Effect of NAM on *CUP1* copy number. Similar to Figure 1B (*n* = 3 or more biological replicates). Statistical significance of copy number alteration was examined between day 0 and day 5 using *t*-test (*P< 0.05).

To further investigate this phenomenon, we constructed a strain in which dCas9 can be induced using β-estradiol without affecting cell growth (Figure 1A and Supplementary Figure S1C). This strain utilizes the artificial transcription factor GEV (Gal4 DNA-binding domain, estrogen receptor and VP16 transcription activation domain) (22), which translocates to the nuclei upon binding to β-estradiol and activates the *GAL1* promoter to induce dCas9 expression. We grew the strain with daily dilution of the culture with fresh medium, extracted genomic DNA at various time points after β-estradiol addition, and measured the *CUP1* copy number by qPCR. When *TEF1*-targeted dCas9 was induced, the copy number (∼16 copies) did not show any significant change throughout the experiment (Figure 1B). In contrast, induction of *CUP1-*targeted dCas9 rapidly decreased the copy number in a time-dependent manner (Figure 1B). The extent of decrease varied from one sgRNA to another and was enhanced by the concurrent expression of three sgRNAs (*CUP1*a+b+c) (Figure 1B). The rate of decrease gradually slowed down, and the copy number appeared to reach a plateau in an extended culture (Supplementary Figure S1D).

As all three abovementioned *CUP1* sgRNAs (*CUP1*a, b and c) bind to the same DNA strand, we designed an sgRNA that binds to the opposite strand (*CUP1*d) to test whether dCas9 reduces the *CUP1* copy number in a strand-specific manner. *CUP1*d reduced the copy number with an efficiency largely comparable to that of *CUP1*a (Figure 1C). When combined, the two sgRNAs accelerated the copy number reduction (Figure 1C). These results suggested that dCas9 targeted to either DNA strand likely reduces the *CUP1* copy number.

We next sought to determine whether the effect described above was specific to the *CUP1* array. The yeast genome has several tandem repeats other than the *CUP1* array, including the *ENA1* array encoding P-type ATPase sodium pumps (40). The *ENA1* array comprises a tandem array of three paralogous genes in the S288c reference genome sequence, namely *ENA1*, *ENA2* and *ENA5*, but some strains harbor four or more paralogs (34,41). It was determined by nanopore sequencing that the strain used in this study had five paralogs (Figure 1D and Supplementary Figure S1E). We designed three sgRNAs for *ENA1* to examine whether dCas9 targeting affects the copy number of *ENA1* paralogs (Figure 1D). When dCas9 was targeted to *ENA1* (*ENA1*a+b+c), the copy number decreased slowly (Figure 1E). This decrease was apparent in the presence of nicotinamide (NAM) (Figure 1E, see below).

Taken together, these results demonstrated that when targeted to tandem repeats, dCas9 reduces the copy number of repeat units in a sequence-specific manner.

### NAM accelerates dCas9-induced copy number reduction of tandem repeat units

A previous study reported that NAM induces *CUP1* CNV (16). NAM is an inhibitor of the NAD^+^-dependent histone deacetylase family, which includes Sir2, Hst1, Hst2, Hst3 and Hst4. Accordingly, concurrent deletion of *SIR2*, *HST3* and *HST4* destabilized the *CUP1* array (16). Conversely, deletion of *RTT109*, encoding the sole histone acetyltransferase responsible for histone H3 acetylated at Lys-56 (H3K56ac), suppressed the NAM-induced CNV (16). In our study, the effect of NAM on the *CUP1* copy number was not significant in the control strain with *TEF1*-targeted dCas9 (Figure 1F). In contrast, NAM substantially accelerated copy number reduction in the presence of *CUP1*-targeted dCas9 (Figure 1F). NAM also accelerated the copy number reduction of *ENA1* paralogs induced by *ENA1*-targeted dCas9 (Figure 1E). Consistent with the previous study (16), NAM failed to exert its effect on dCas9-induced *CUP1* CNV in the absence of Rtt109 (Supplementary Figure S1F). These results suggest that NAM enhances dCas9-induced destabilization of tandem repeats through the elevation of H3K56ac.

### Binding of a single dCas9 molecule can destabilize the *CUP1* array

**Figure 2.**
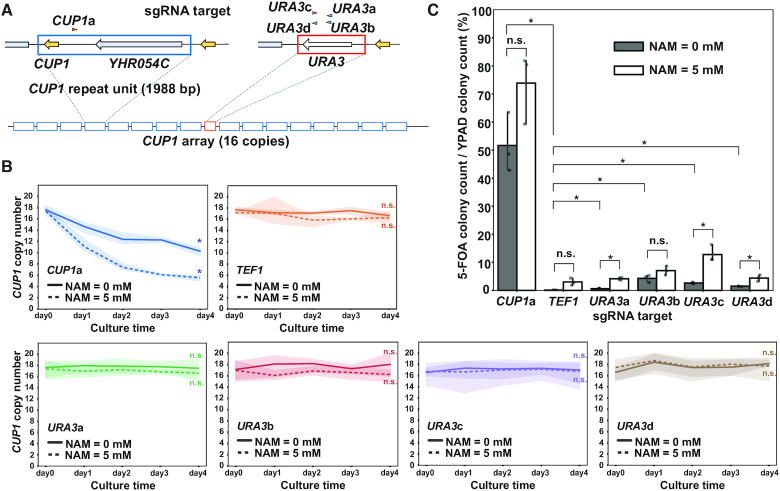
*CUP1* array destabilization by single molecules of dCas9. (**A**) Schematic of *URA3*-bearing *CUP1* array. Arrowheads indicate sgRNAs for *CUP1* and *URA3* (orange, Watson strand; blue, Crick strand). (**B**) Time course of *CUP1* copy number in the strain with the *URA3*-bearing *CUP1* array in the presence or absence of NAM. Similar to Figure 1B (*n* = 3 biological replicates). Statistical significance of copy number alteration was examined between day 0 and day 4 using *t*-test (*P< 0.05). (**C**) Frequency of *URA3* loss. Following four-day induction of dCas9 with the indicated sgRNAs in the presence or absence of 5 mM NAM, cells were spread on agar plates supplemented with or without 5-FOA. Data are represented as mean ± standard deviation (*n* = 3 biological replicates). Statistical significance was examined between *TEF1*-targeted strain and each of the other strains and between the conditions with and without NAM in each strain using *t*-test (*P< 0.05).

We wondered whether binding of a single dCas9 molecule can affect the copy number of tandem repeat units. To address this issue, we deployed a classical genetic assay based on the loss of *URA3* integrated into the *CUP1* array. For this assay, we generated a strain carrying a *URA3* cassette in the center of the *CUP1* array comprising 16 repeat units (Figure 2A and Supplementary Figure S2A). Upon destabilization of the *CUP1* array, a fraction of recombination events between the repeat units led to the loss of the *URA3* cassette, conferring on cells resistance to 5-fluoro-orotic acid (5-FOA). A four-day induction of *CUP1*-targeted dCas9 reduced the average *CUP1* copy number (Figure 2B). Following this, we spread the cells onto agar plates supplemented with or without 5-FOA. As expected, the *CUP1*-targeted strain contained more 5-FOA-resistant cells than the control *TEF1*-targeted strain (51.7% versus 0.1%, 374.9-fold) (Figure 2C).

Confirming the performance of the genetic assay with *CUP1*-targeted dCas9, we next tested whether *URA3*-targeted dCas9 destabilizes the *CUP1* array. We used four sgRNAs (*URA3*a, b, c and d) to generate four strains with *URA3*-targeted dCas9 and subjected them to both the qPCR and genetic assays (Figure 2A). The qPCR assay failed to detect any significant decrease in the average *CUP1* copy number in the four strains, presumably because copy number reduction occurred only in a limited fraction of the cell population (Figure 2B). However, in the genetic assay, the *URA3*-targeted strains generated 5-FOA-resistant clones much more frequently than the control *TEF1*-targeted strain (*URA3*a, 0.6%; *URA3*b, 4.3%; *URA*3c, 2.6%; *URA3*d, 1.4%; *TEF1*, 0.1%) (Figure 2C). Point mutations of *URA3* could also confer 5-FOA resistance, and dCas9 was reported to induce base substitutions and indels (3). However, qPCR using DNA isolated *en masse* from 5-FOA resistant colonies confirmed deletion of *URA3* cassette, indicating that the contribution of point mutations was negligible (Supplementary Figure S2B). We thus concluded that even a single molecule of dCas9 can destabilize the *CUP1* array, albeit much less efficiently than multiple dCas9 molecules targeted to individual repeat units.

### dCas9 both contracts and expands the *CUP1* array

**Figure 3.**
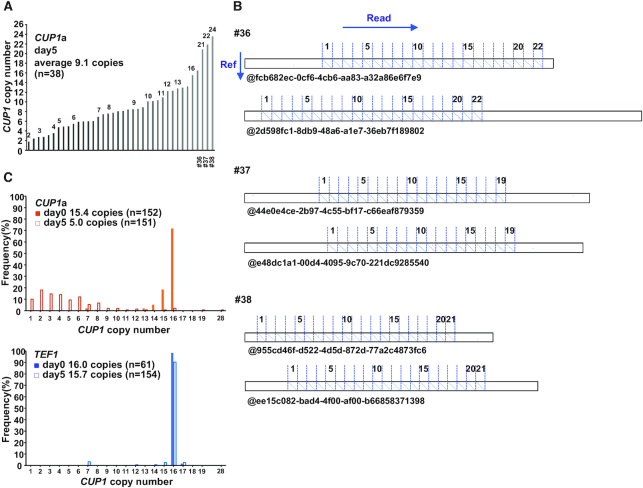
dCas9-induced expansion of *CUP1* array. (**A**) *CUP1* copy numbers in individual clones. Single colonies isolated at day 5 of dCas9 induction (Figure 1B) were used for qPCR. (**B**) *CUP1* array structure revealed by nanopore sequencing. DNAs prepared from clones #36, #37 and #38 in Figure 3A were sequenced with MinION. Dot plots were generated between the reference sequence of *CUP1* repeat unit (vertical axis) and nanopore sequencing reads (horizontal axis). Two representative reads are shown for each clone. (**C**) Population structure of *CUP1* array. DNAs prepared from the cells with the indicated sgRNAs at days 0 and 5 of dCas9 induction (Figure 1B) were sequenced with MinION. *CUP1* copy number determined from dot plots are shown.

The qPCR assay using an aliquot of liquid culture revealed the population average copy number but did not demonstrate the cell-to-cell variation. To determine this variation, we isolated single colonies from the cells cultivated in a liquid medium supplemented with β-estradiol and determined the *CUP1* copy number of each clone by qPCR (Figure 3A). As expected from the decreased population average, most of the 38 clones examined had reduced copy numbers. However, three clones appeared to have higher copy numbers than the original strain (#36–#38, Figure 3A).

The observed increase in the *CUP1* copy number does not necessarily indicate the expansion of *CUP1* array, as the *CUP1* repeat unit has been shown to exist as an extrachromosomal circular DNA (42). Furthermore, aneuploidy may include chromosome VIII bearing the *CUP1* array. We thus performed long-read sequencing using the MinION nanopore sequencer to reveal the *CUP1* array structure in the three clones (Figure 3A). We selected reads containing both 5′- and 3′-flanking regions of the *CUP1* array (i.e. reads spanning the entire array), generated a dot plot between each read and the reference sequence of *CUP1* repeat unit, and manually counted the number of units from each plot (Figure 3B). We also developed a Fourier transform-based algorithm to calculate the copy number directly from nanopore reads to validate the results of manual counting (Supplementary Figure S3A). Consequently, the clones #36, #37 and #38 were demonstrated to harbor *CUP1* arrays composed of 22, 19 and 21 repeat units, respectively (Figure 3B).

We next examined the population structure of the *CUP1* array by sequencing genomic DNA prepared from *CUP1*- and *TEF1*-targeted strains at days 0 and 5 of induction. In the *TEF1*-targeted strain, the *CUP1* copy number in the array did not change during the culture (16.0 and 15.7 copies on average at days 0 and 5, respectively, by both manual and computational counting) (Figure 3C and Supplementary Figure S3B). In contrast, in the *CUP1*-targeted strain, the copy number distribution was obviously different between days 0 and 5. The copy number at day 0 showed a relatively homogenous distribution within a range of 14–16 copies (15.4 and 14.9 copies on average by manual and computational counting, respectively). This low level of heterogeneity was presumably attributable to leaky expression of dCas9, as it was observed only in the presence of *CUP1* sgRNA. The copy number at day 5 exhibited a significantly heterogenous distribution (5.0 and 4.7 copies on average by manual and computational counting, respectively) (Figure 3C and Supplementary Figure S3B). While 16 reads contained only a single copy of the *CUP1* repeat unit, two reads spanned arrays comprising 19 and 28 units (Supplementary Figure S3C). Note that longer arrays are underrepresented in population analysis by nanopore sequencing compared to that by qPCR, as a longer array will have fewer reads spanning the entire array (Supplementary Figure S3D). Nevertheless, nanopore sequencing unequivocally demonstrated the expansion of the *CUP1* array. Interestingly, it also revealed *CUP1* arrays with interstitial deletions (Supplementary Figure S3C), which led to the slight difference between the copy numbers estimated by manual and computational counting (Figure 3C and Supplementary Figure S3B).

Taken together, dCas9 contracts and expands the *CUP1* array in the majority and minority of cells, respectively, thereby inducing heterogeneity in the array structure.

### dCas9 blocks replication fork progression in vivo

**Figure 4.**
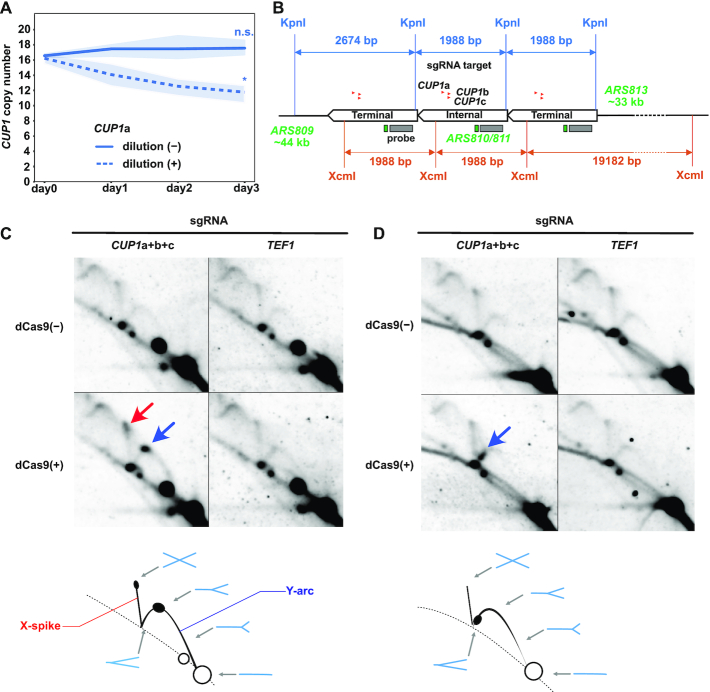
dCas9-mediated impairment of replication fork progression. (**A**) Effect of cell proliferation on dCas9-induced reduction of *CUP1* copy number. Similar to Figure 1B, except that saturated cultures without daily dilution are included (*n* = 3 biological replicates). Statistical significance of copy number alteration was examined between day 0 and day 3 using *t*-test (*P< 0.05). (**B**) Design of 2D-AGE experiments. The *CUP1* array map indicates the positions of KpnI and XcmI restriction sites, sgRNAs, hybridization probe, ARSs and the lengths of bands detectable with the probe. Only a single copy of internal repeat units is depicted for clarity. (**C**) Representative 2D-AGE images of KpnI-digested DNA fragments. Blue and red arrows indicate the spots on the Y-arc (stalled replication fork) and the X-spike (highly branched X-shaped molecule), respectively. Schematics at the bottom indicate the interpretation of 2D-AGE patterns. Exposure time for signal detection was 4 h. (**D**) Representative 2D-AGE images of XcmI-digested DNA fragments. Blue arrow indicates the spot on the Y-arc. Schematics at the bottom indicate the interpretation of 2D-AGE patterns. Exposure time for signal detection was 4 h.

We hypothesized that dCas9-induced destabilization of the *CUP1* array stems from dCas9-mediated impairment of replication fork progression. Indeed, *CUP1*-targeted dCas9 failed to alter the copy number when the culture was saturated to terminate DNA replication (Figure 4A). To test this hypothesis directly, we conducted neutral-neutral 2D-AGE (43) and analyzed the status of DNA replication intermediates including the *CUP1* repeat unit by Southern blot hybridization (Figure 4B).

Prominent spots appeared on Y-arcs upon the induction of *CUP1*-targeted dCas9 (Figure 4C and D, blue arrows). These results indicated that dCas9 induced replication fork stalling in the *CUP1* unit. When KpnI-digested fragments were analyzed, the spot was observed approximately at the apex of Y-arc, indicating replication fork stalling around the midpoint of the restriction fragment. Moreover, another spot appeared at the tip of X-spike, suggesting an accumulation of highly branched X-shaped molecules (Figure 4C, red arrow). It could be interpreted as two replisomes colliding around the midpoint of the fragment. When XcmI-digested fragments were analyzed, a spot was detected in the descending part of Y-arc upon induction of *CUP1*-targeted dCas9 (Figure 4D), suggesting replication fork stalling near an end of the fragment. Considering the dCas9-bound sites in the XcmI fragment, we speculate that the stalled replication fork likely originated from *ARS813*, located ∼33-kb upstream of the *CUP1* array (Figure 4B). Although each *CUP1* repeat unit has a weak replication origin (*ARS810/811*) (44,45), we failed to observe the bubble-arc corresponding to the DNA replication bubble. This was presumably because *ARS810/811* fires much less frequently than the closest neighboring replication origin for the *CUP1* array. In summary, dCas9 impairs replication fork progression in the vicinity of its binding sites.

### The RPC, accessory helicase and recombination proteins modulate dCas9-induced *CUP1* CNV

**Figure 5.**
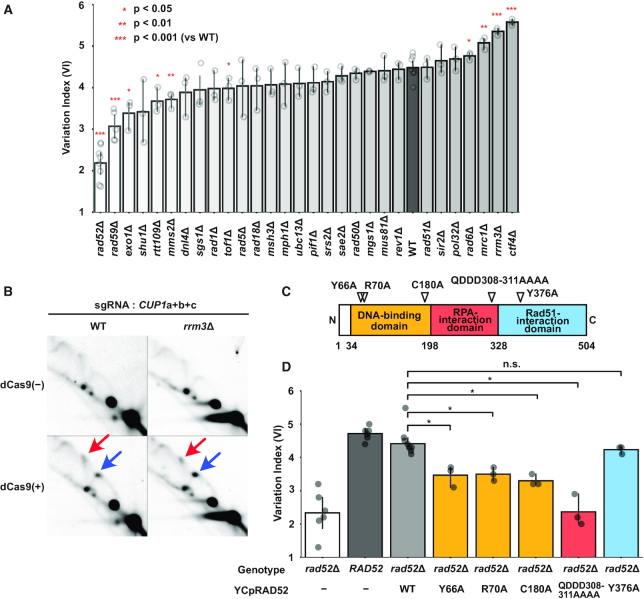
Genetic analysis of genes involved in dCas9-induced *CUP1* CNV. (**A**) Alteration of *CUP1* copy number in mutant strains. Each strain with three sgRNAs (*CUP1*a+b+c) was cultivated for two days in the presence of β-estradiol. Percentile change of *CUP1* copy number estimated by qPCR was divided by the number of cell division estimated from absorbance to calculate VI. Data are represented as mean ± standard deviation (*n* = 3 or more biological replicates). Statistical significance was calculated using *t*-test compared to the wild-type (WT) strain (*P< 0.05; **P < 0.01; ***P < 0.001). (**B**) Replication fork stalling in the wild-type and *rrm3*Δ strains. 2D-AGE patterns of KpnI-digested DNA are shown for the WT and *rrm3*Δ strains. Blue and red arrows indicate the spots on the Y-arc (stalled replication fork) and the X-spike (highly branched X-shaped molecule), respectively. The samples of the wild-type strain are identical to those in Figure 4C but exposure time for signal detection was 1 h. (**C**) Schematic of Rad52 domain structure with separation-of-function mutations. (**D**) Suppression of defective dCas9-induced *CUP1* CNV in the *rad52*Δ strain by wild-type and separation-of-function alleles. Data are represented as mean ± standard deviation (*n* = 3 or more biological replicates). Statistical significance was examined between the strains harboring the WT and the separation-of-function alleles on YCpRAD52 using *t*-test (*P< 0.05).

A stalled replication fork may either resume progression or collapse. In the latter case, the cell exploits recombinational repair pathways to rescue the collapsed fork. We hypothesized that dCas9-induced destabilization of tandem repeats is attributable to a repair process of replication forks stalled by dCas9. To obtain genetic evidence for and mechanistic insights into this process, we evaluated dCas9-induced reduction of the *CUP1* copy number in a series of strains in which genes involved in replication fork stability and major DNA damage repair pathways were deleted (Figure 5A and Supplementary Figure S4A–C). We used qPCR to estimate the *CUP1* copy number at days 0 and 2 of *CUP1*-targeted dCas9 induction. To normalize the effects of differential growth rates among the strains, we evaluated the effects of gene deletions using a variation index (VI) defined as copy number change (%) per cell division (Supplementary Figure S4A, B). None of the 29 deletants examined abolished the dCas9-induced copy number alteration. The response to dCas9-mediated replication fork stalling may thus be redundant, with one pathway likely serving as a back-up for another. Nevertheless, certain strains showed significant change in VI (Figure 5A).

A significantly increased VI was observed in strains with *CTF4, RRM3* and *MRC1* deletions (*ctf4*Δ, 5.57, *P* = 1.3E-06; *rrm3*Δ, 5.35, *P* = 1.0E–05; *mrc1*Δ, 5.08, *P* = 0.001; WT, 4.48) (Figure 5A). Although these genes have been implicated in tandem repeat stabilization (14–18), the *CUP1* copy number was stably maintained in the *ctf4*Δ, *rrm3*Δ and *mrc1*Δ strains with *TEF1*-targeted dCas9 (Supplementary Figure S4D). Note that Ctf4 and Mrc1 are components of the RPC and are known to affect fork stability (46–48). The accessory helicase Rrm3 removes obstacles in front of the replisome (17,49). Consistently, it appeared that dCas9-induced replication fork stalling was enhanced in the *rrm3*Δ strain compared to the wild-type strain (Figure 5B and Supplementary Figure S4E–G). Compared to these three strains, the increment of VI in the *rad6*Δ strain was less significant (4.76, *P* = 0.014). Although the *pol32*Δ strain was reported to accelerate *CUP1* array contraction (16), it failed to exert a significant effect on the dCas9-induced CNV (4.69, *P* = 0.173).

A significantly decreased VI was observed in strains with *RAD52* and *RAD59* deletions (*rad52*Δ, 2.19, *P* = 6.62E–09; *rad59*Δ, 3.07, *P* = 7.31E–05). Notably, Rad52 plays critical roles in various recombinational repair pathways, and Rad59 is paralogous to and cooperates with Rad52. We thus used Cas12a-mediated gene editing (25) to disrupt *RAD52* and *RAD59* in the *rad59*Δ and *rad52*Δ strains, respectively. These double mutants failed to show a significantly decreased VI compared to the *rad52*Δ strain, suggesting that *RAD52* is epistatic to *RAD59* (Supplementary Figure S5A–D). Compared to the *rad52*Δ and *rad59*Δ strains, the decrease of VI was less significant in strains with *EXO1*, *RTT109*, *MMS2* and *TOF1* deletions (*exo1*Δ, 3.39, *P* = 0.010; *rtt109*Δ, 3.67, *P* = 0.011; *mms2*Δ, 3.72, *P* = 0.002; *tof1*Δ, 3.98, *P* = 0.030).

These results collectively indicated critical roles for the RPC components, accessory helicase, and recombination proteins in dCas9-induced *CUP1* array contraction.

### dCas9-induced *CUP1* CNV involves SSA by Rad52

As *RAD52* deletion had the largest impact on the VI (Figure 5A and Supplementary Figure S5A), we sought to determine how Rad52 contributes to dCas9-induced *CUP1* array contraction. To this end, we took advantage of reported separation-of-function *rad52* alleles. Rad52 is composed of three domains (Figure 5C). The N-terminal domain is evolutionarily conserved and mediates interactions with DNA, Rad52 (self-oligomerization), and Rad59. Class C mutants bearing mutations in this domain (*rad52-Y66A*, *-R70A*, *-W84A*, *-R85A*, *-Y96A*, *-R156A*, *-T163A*, *-C180A* and *-F186A*) are defective in SSA but proficient in HR (50–52). The central domain is required for binding to the single-stranded DNA (ssDNA)-binding protein RPA and formation of the Rad52 repair center. A mutant allele of this domain, *rad52-QDDD308*-*311AAAA*, encodes a protein that is defective in RPA-binding and mediator activity to exchange RPA for Rad51 on ssDNA, but is proficient in DNA binding, Rad51 binding, and SSA *in vitro* (53). The Rad52 C-terminal domain is also required for mediator activity. A mutant allele of this domain, *rad52-Y376A*, encodes a protein incapable of binding to Rad51 (54–56).

The wild-type and separation-of-function alleles were expressed in the *rad52*Δ strain under the control of the *RAD52* promoter on a centromeric plasmid vector. Immunoblot analysis confirmed comparable expression levels among the wild-type and mutant proteins except for Rad52-C180A (Supplementary Figure S5E). The *RAD52* allele suppressed defects in the *rad52*Δ strain, elevating the VI to a level comparable to that of the wild-type strain (Figure 5D and Supplementary Figure S5F–H). However, class C mutant alleles (*rad52-Y66A*, -*R70A* and -*C180A*) only partially suppressed the defect, indicating the involvement of the N-terminal domain and hence SSA (Figure 5D and Supplementary Figure S5F–H). Intriguingly, the *rad52-QDDD308–311AAAA* allele barely suppressed the defect (Figure 5D and Supplementary Figure S5F–H). In contrast, the *rad52-Y376A* suppressed the defect as efficiently as *RAD52*, demonstrating the dispensability of Rad51 binding and hence mediator activity (Figure 5D and Supplementary Figure S5F–H). Interestingly, depletion of Rad59, a C-terminally-truncated Rad52 paralog with SSA but not mediator activity, exerted a decelerating effect second only to that of Rad52 (Figure 5A). Furthermore, Rad51 depletion did not affect destabilization (Figure 5A). Thus, the data on the separation-of-function alleles were consistent with the deletant data.

Taken together, Rad52 likely contributes to dCas9-induced destabilization of tandem repeats through its involvement in the annealing of RPA-coated ssDNA, but not via its mediator activity.

## DISCUSSION

### dCas9 as a replication fork barrier

The highly plastic nature of the *CUP1* array structure has been attracting attention for its role in environmental adaptation. A previous study reported critical roles for promoter activity and H3K56ac in *CUP1* CNV (16). Here we showed that dCas9 induces CNV in the *CUP1* and *ENA1* arrays (i.e., array contraction and expansion), especially in the presence of NAM (Figures 1 and 3). Notably, dCas9 rapidly decreased the copy number even in the absence of transcriptional *CUP1* induction (Figure 1). Although *RTT109* deletion stabilizes the *CUP1* array even in the presence of active transcription (16), *CUP1*-targeted dCas9 still destabilized the array in the *rtt109*Δ strain (Figure 5A). These results imply a high efficiency of dCas9 in inducing focal genomic instability. To our knowledge, this study is the first demonstration of dCas9-induced SVs. Intriguingly, NAM enhanced dCas9-induced *CUP1* CNV in a H3K56ac-dependent manner (Supplementary Figure S1F). Since H3K56ac regulates replication-coupled nucleosome assembly (57), its hyper-elevation likely alters chromatin status, thus affecting the stability of stalled replication fork and the accessibility of recombinational repair proteins and/or dCas9. These possibilities remain to be examined in future studies.

As both transcription and dCas9 induce *CUP1* CNV, dCas9 may serve as a mimic of RNA polymerase through its R-loop formation, with dCas9 generating an even longer R-loop than RNA polymerase. Notably, R-loop formation is a major threat to genome stability (58). We thus hypothesized that dCas9 and replisomes induce a conflict similar to that observed between transcription and replication. Consistent with this scenario, we found that dCas9 impedes replication fork progression in vivo (Figure 4). Interestingly, a recently published report demonstrated the ability of dCas9 to block replisome progression in vitro (59).

Proteins tightly bound to DNA can impair replication fork progression, and some of them serve as physiological blocks (60). Examples of fork-blocking proteins include Tus binding to the replication terminator *Ter* of *Escherichia coli*, Fob1 binding to the replication fork barrier in rDNA of budding yeast, and Rtf1 binding to *RTS1* in the mating locus of the fission yeast *Schizosaccharomyces pomb*e (60). While these proteins function in an orientation-dependent manner, dCas9 appears to block the replication fork approaching from either side in vivo (Figure 1), consistent with the previous finding in vitro (59). The accumulation of highly branched X-shaped molecules at the tip of X-spike in 2D-AGE appeared to be consistent with replication fork stalling at both sides of dCas9-bound sites, although we should note that it may also reflect entanglements between sister chromatids (Figure 4C). It remains to be seen in future studies whether dCas9 is equally effective in blocking the replication fork approaching from either direction.

Aside from the professional fork-blockers, proteins such as LacI and TetR can impair replication fork progression when bound to highly iterated arrays of *lacO* and *tetO*, respectively (60). We investigated whether even a single molecule of dCas9 can serve as a sufficiently strong barrier to induce genomic instability. To address this issue, we repurposed the classical genetic assay using a *URA3*-bearing *CUP1* array to sensitive detection of focal genomic instability induced by a single molecule of *URA3*-targeted dCas9 (Figure 2). The results showed that the binding of even a single molecule of dCas9 can destabilize the array. If each dCas9 molecule independently destabilizes the *CUP1* array, then targeting of dCas9 to each repeat unit multiplies the destabilizing effects, thereby leading to rapid contraction of the array.

Whether the replisome and dCas9 collide on the genome and have a mutual contact remains unclear. As the replisome approaches dCas9 in vivo, the torsional stress between their binding sites likely increases and may finally prevent replisome progression. This could explain why 2D-AGE indicated replication fork stalling around the midpoint of KpnI fragment, although the dCas9-bound sites were slightly off-centered (Figure 4). A higher-resolution method for mapping the position of the stalled replication fork is required to address this issue in future studies.

### Cellular responses to dCas9-mediated replication fork stalling

When the replication fork encounters with an obstacle on DNA, the stability of the former and the removal of the latter should be critical. Ctf4 was demonstrated to protect arrested replication forks against breakage to suppress genome rearrangements, including hyper-amplification of rDNA (47). Mrc1 interacts with Tof1–Csm3 to form the heterotrimeric fork protection complex. Deletion of *CTF4* and *MRC1* significantly accelerated dCas9-induced reduction of the *CUP1* copy number (Figure 5A). In the *ctf4*Δ and *mrc1*Δ strains, dCas9-mediated replication fork stalling appeared to be diminished compared to the wild-type strain (Supplementary Figure S4E–G), presumably reflecting the breakage of destabilized replication forks. The accessory helicase Rrm3 is responsible for the removal of obstacles in front of the replisome. In the *rrm3*Δ strain, the VI was significantly increased and dCas9-mediated replication fork stalling appeared to be enhanced (Figure 5A, B and Supplementary Figure S5G). Consistently, depletion of Tof1, which counteracts Rrm3, resulted in a modest but significant decrease of the VI (Figure 5A). These data collectively underscored the importance of replisome protection and dCas9 removal in tandem repeat stability.

Despite the activities for fork protection and obstacle removal, stalling is occasionally prolonged to result in fork collapse. Cells have various mechanisms to cope with collapsed forks, which likely induce *CUP1* CNV. Our genetic analysis indicated that *RAD52* and its paralog *RAD59* have the largest and second-largest contributions to *CUP1* CNV, respectively (Figure 5A). Genetic analysis using separation-of-function alleles indicated that Rad52 destabilizes tandem repeats via its SSA activity, but not its mediator activity to exchange RPA for Rad51 (Figure 5D). Although Rad52 and Rad59 mediate SSA, Rad52 but not Rad59 can perform this function in the presence of RPA (61). Based on the result of *rad52* allele encoding a protein defective in RPA binding, we assumed that Rad52 mediates the annealing of RPA-coated ssDNA. Note that Rad52 was dispensable to restore rDNA copy number in the absence, but not the presence, of histone chaperone Asf1 (62). It would be intriguing to examine whether the requirement of Rad52 for dCas9-induced *CUP1* CNV is mitigated in the absence of Asf1.

Conventional SSA occurs after DSB and subsequent end resection. However, qPCR failed to provide evidence for DSB around dCas9-bound sites. Similarly, time-lapse imaging failed to reveal significant difference in Rfa1 focus formation, indicative of DSB, between the strains with *CUP1*-targeted dCas9 and with no sgRNA. Thus, we have so far not obtained clear evidence for dCas9-induced DSB. The decease of VI in the *exo1*Δ strain defective in end resection was less significant compared to the *rad52*Δ and *rad59*Δ strains (Figure 5A). It is conceivable that dCas9 induces destabilization without forming prominent DSBs, such as those generated by replication fork breakage. In this context, a new mechanism termed inter-fork strand annealing (IFSA) has attracted our attention. IFSA explains the inter-repeat recombination induced by Rtf1/*RTS1* system in fission yeast, involves Rad52 and Exo1 but not Rad51, and occurs without replication fork breakage (63). An IFSA-like mechanism may operate in budding yeast to mediate dCas9-induced CNV of tandem repeat units. Alternatively, tandem repeat structure may help SSA-mediated DSB repair to proceed too quickly to be detected with conventional approaches. Interstitial deletions occasionally found by nanopore sequencing may indicate at least a limited involvement of DSB in copy number alterations (Supplementary Figure S3C).

Less prominent but significant effects on destabilization were observed in the *mms2*Δ and *rad6*Δ strains (Figure 5A). Both *MMS2* and *RAD6* encode components of the error-free TS pathway (64). However, the *mms2*Δ and *rad6*Δ strains exerted mutually opposite effects. Moreover, depletion of the other components of this pathway (Rad18, Ubc13 and Rad5) failed to have significant effects on VI. Since all these proteins are involved in ubiquitination of proliferating cell nuclear antigen (PCNA), it is intriguing to examine the ubiquitination-defective PCNA mutant (Pol30-K146R). In anyway, we assumed that TS has little if any contribution to dCas9-induced tandem repeat destabilization. Similarly, HR, NHEJ, BIR and TLS did not appear to play major roles because no significant change of VI was observed in the *rad51*Δ, *dnl4*Δ, *pol32*Δ and *rev1*Δ strains, respectively (Figure 5A).

Taken together, it remains to be seen in future studies how Rad52 and Rad59 mediate the cellular response to dCas9-mediated stalling of replication fork. It is also intriguing to examine the response in other species, including mammals, in which the preference in the choice of recombinational repair pathways may be different from that in the budding yeast.

### Potential risk and application of dCas9-mediated replication fork stalling

This work has identified a potential risk of dCas9, distinct from the previously reported mutagenicity of the R-loop (3). For instance, for the sake of sensitivity, live-cell imaging studies often target dCas9 fused or complexed with fluorescent proteins to tandem repeats. Extended cultivation of such cells may result in contraction of the targeted tandem repeats, leading to not only compromised sensitivity but also an unexpected outcome. Even at a single-copy target site, dCas9 can impede replication fork progression and may thus induce SVs. This is especially true when recombinogenic genomic features are present around the target site, as was in the case for the *URA3* cassette integrated in the *CUP1* array (Figure 2). In this context, it is intriguing to note that the results of our genetic analysis (Figure 5) suggests a potential utility of Rad52 inhibitors (65,66) in reducing the risk of dCas9-induced focal genomic instability, albeit at the expense of general defects in various types of recombination.

Conversely, our findings imply that dCas9 provides a versatile tool for impeding replication fork progression at the genomic site of interest in vivo. Indeed, controlled replication fork stalling can accelerate mechanistic studies on genome stability. For this purpose, the Tus/*Ter*-system has been successfully used in both yeast and mammalian cells (67,68). However, this system requires its users to integrate *Ter* sequences into the regions of interest. In contrast, dCas9 is readily targetable to virtually any genomic regions by simply designing appropriate sgRNAs. Moreover, since dCas9-mediated replication fork stalling works without modifying the genomic sequence, it would enable recapitulation of natural SV generation, thus providing a novel approach for modeling evolution and pathogenesis. Initial amplification of a single-copy gene likely involves mechanisms such as re-replication-induced gene amplification (69) and origin-dependent inverted repeat amplification (70). In both mechanisms, the borders of amplified regions are defined by the positions of replication fork collapse. We therefore expect that dCas9-mediated replication fork stalling provides a versatile tool to manipulate SVs including gene duplication, a critical driver of evolution.

## DATA AVAILABILITY

The source code of DNA Seq Detector used in this study is available at GitHub (https://github.com/poccopen/DNA_Sequence_Detector). Nanopore sequencing data used in this study were deposited in DRA under accession number DRA010708.

## Supplementary Material

gkaa1241_Supplemental_Files
