# Supplementary material for: Catalytically inactive Cas9 impairs DNA replication fork progression to induce focal genomic instability
Source: Nucleic Acids Res. 2021 Jan 4;49(2):954–68. doi: 10.1093/nar/gkaa1241 (PMC7826275; doi:10.1093/nar/gkaa1241)
Supplement: gkaa1241_Supplemental_Files [file gkaa1241_supplemental_files.zip › Supporting Information.pdf]

Supporting Information

Catalytically inactive Cas9 impairs DNA replication fork progression to induce focal genomic instability

Goro Doi[1], Satoshi Okada[1], Takehiro Yasukawa[2], Yuki Sugiyama[1], Siqin Bala[1], Shintaro Miyazaki[3], Dongchon Kang[2] and Takashi Ito[1,*]

SUPPLEMENTARY FIGURE LEGENDS

Supplementary Figure S1. dCas9-induced copy number reduction of tandem repeat units.

(A) *CUP1* copy number of strains with constitutive expression of *CUP1*- and *TEF1*-targeted dCas9. Both strains use *SNR52* and *CSE4* promoters to constitutively express sgRNA and dCas9, respectively. Data are represented as the mean of two biological replicates.

(B) Time course of *CUP1* copy number of the strains used in Supplementary Figure S1A.

(C) Effect of dCas9 induction on cell growth. Growth in the presence of 0, 10, and 100 nM β-estradiol were examined for the strains using *CUP2* or *ACT1* promoter for GEV expression (n = 1). Doubling time was determined from the growth curve drawn based on optical density measured every 10 min.

(D) Time course of *CUP1* copy number. Similar to Figure 1B, except that culture was extended to day 9 (n = 1).

(E) Dot plots of a nanopore sequencing read spanning the entire *ENA1* array. Top, self dot plot for the read. Bottom, dot plot between the *ENA1* reference sequence of *ENA1* (vertical axis) and the read (horizontal axis).

(F) Effects of NAM on dCas9-induced *CUP1* copy number reduction in the WT and *rtt109*Δ strains. Similar to Figure 1B (n = 3 or more biological replicates).

Supplementary Figure S2. Destabilization of *URA3*-bearing *CUP1* array by *URA3*-targeted dCas9.

(A) Dot plots between a nanopore sequencing read spanning the *URA3*-bearing *CUP1* array (horizontal axis) and the reference sequence of *CUP1* repeat unit (RU) or *URA3* cassette (vertical axis).

(B) qPCR of *CUP1* and *URA3* in DNA isolated *en masse* from 5-FOA-resistant colonies.

Supplementary Figure S3. dCas9-induced expansion of *CUP1* array.

(A) Computational counting of *CUP1* repeat units in nanopore reads. Boxed are the tandemly iterated regions identified by DNA Sequence Detector software. Dot plots between the reads and the reference sequence of *CUP1* repeat unit are shown on the top. Note that the software correctly identifies the tandemly iterated regions from a nanopore read.

(B) Population structure of the *CUP1* array. Similar to Figure 3C, except that copy numbers are computationally counted with DNA Sequence Detector.

(C) Various structures of *CUP1* array revealed by nanopore sequencing. Dot plots between the *CUP1* reference sequence (vertical axis) and individual reads (horizontal axis) are shown. Arrows indicate the positions of interstitial deletions.

(D) Histogram of nanopore read lengths after log transformation. Green vertical lines indicate the length of the wild-type *CUP1* array composed of 16 repeat units (~32 kb).

Supplementary Figure S4. Genetic analysis of genes involved in dCas9-induced *CUP1* CNV.

(A) Percentile decrease of *CUP1* copy number in the strains used in Figure 5A.

(B) Number of cell division in the strains used in Figure 5A.

(C) Initial *CUP1* copy number at day 0 in the strains used in Figure 5A.

(D) *CUP1* copy number change in the WT, *ctf4*Δ, *rrm3*Δ, and *mrc1*Δ strains with *TEF1*-targeted dCas9 (n =2 or more biological replicates).

(E) 2D-AGE images of KpnI-digested DNA fragments of the WT, *rrm3*Δ, *ctf4*Δ, and *mrc1*Δ strains. Panels labeled as WT and *rrm3*Δ #1 are identical to those in Figure 5B. Panels labeled as *rrm3*Δ #2 show biological duplicate data on the *rrm3*Δ strain. Exposure time for signal detection was 1 h.

(F) Strategy for quantitative evaluation of replication fork stalling. 2D-AGE images identical to those in Figure 5B are shown as example. Two images were obtained by exposing the same membrane for 1 h and 1 min. The Stall Spot appeared on the Y-arc in the presence of dCas9 (i.e., stalled replication fork) was quantified from the 1-h exposure image as described in Methods. Similarly, the 1N Spot including the linear ~2-kb fragment was quantified from the 1-min exposure image. We divided the Stall Spot intensity by the 1N Spot intensity on the same membrane, thereby normalizing the amount of DNA prior to comparison among gels.

(G) Quantitative comparison of replication fork stalling among the WT, *rrm3*Δ, *ctf4*Δ, and *mrc1*Δ strains. The Stall Spot was quantified from the gels shown in Supplementary Figure S4E according to the method described in Supplementary Figure S4F. Top panel indicates the simple ratio of Stall Spot against 1N spot, whereas the bottom panel indicates the background-subtracted ratio. The values in each mutant was normalized to that of WT (n =2 or more biological replicates). Note that replication fork stalling was enhanced in *rrm3*Δ but diminished in *ctf4*Δ and *mrc1*Δ strains.

Supplementary Figure S5. Roles for Rad52 in dCas9-induced *CUP1* CNV.

(A) Alteration of *CUP1* copy number in the strains lacking both Rad52 and Rad59. The *rad52*Δ *rad59*Δ strains #1, #2, #3, and #4 (SY7-1, -2, -3, and -4) were generated from the *rad52*Δ strain by Cas12a-mediated gene editing with crRNAs *RAD59*g1, g6, g6, and g8, respectively (Supplementary Table S1 and S4). The *rad59*Δ *rad52*Δ strains #1 and #2 (SY7-6 and -7) were generated from the *rad59*Δ strain by Cas12a-mediated gene editing with crRNA *RAD52*g2 (Supplementary Table S1 and S4). The size and position of deletion in each strain is indicated in Supplementary Table S1. Data are represented as mean ± standard deviation (n = 3 or more biological replicates). Statistical significance was examined between the *rad52*Δ strain and the other strains using t-test (*p<0.05).

(B) Percentile decrease of *CUP1* copy number in the strains used in Supplementary Figure S5A.

(C) Number of cell division in the strains used in Supplementary Figure S5A.

(D) Initial *CUP1* copy number at day 0 in the strains used in Supplementary Figure S5A.

(E) Western blot analysis of Rad52 proteins expressed from a centromeric plasmid in the *rad52*Δ strain. Note that Rad52 proteins were C-terminally FLAG-tagged for the detection with anti-FLAG antibody.

Tubulin-α was used as a loading control for each sample.

(F) Percentile decrease of *CUP1* copy number in the strains used in Figure 5D.

(G) Number of cell division in the strains used in Figure 5D.

(H) Initial *CUP1* copy number at day 0 in the strains used in Figure 5D.

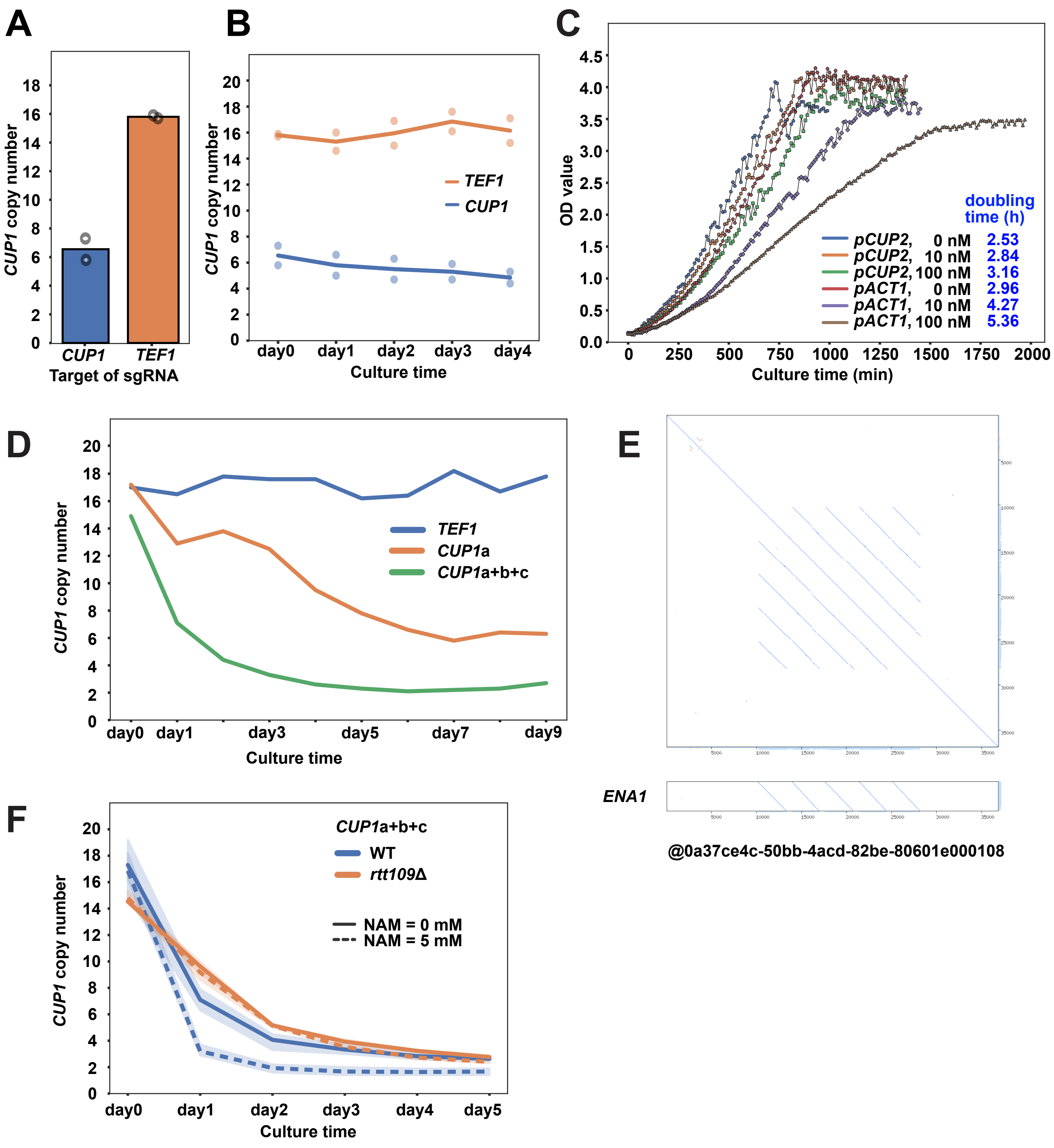

Doi et al., Supplementary Figure S1

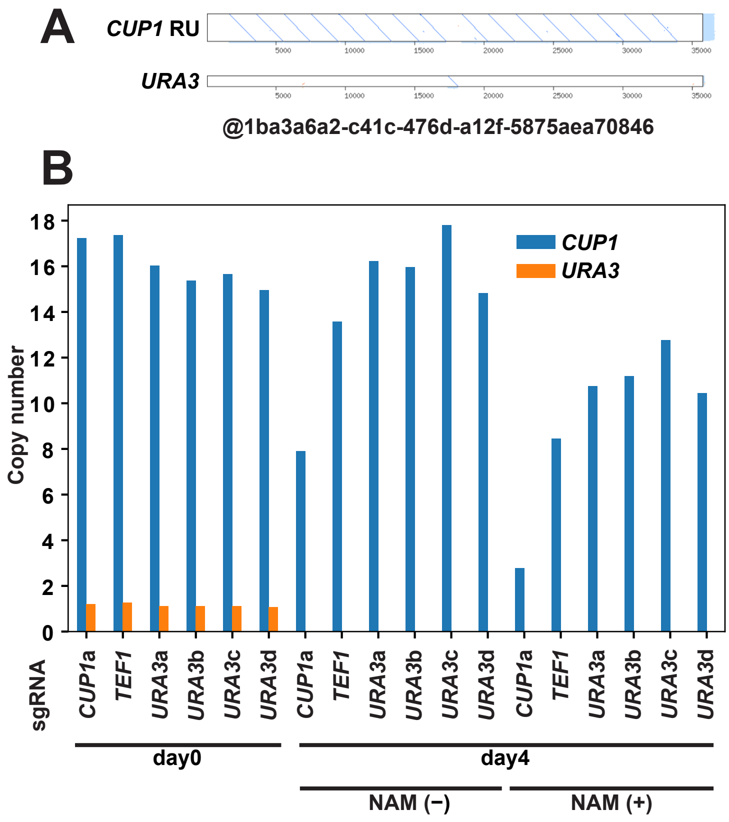

Doi et al., Supplementary Figure S2

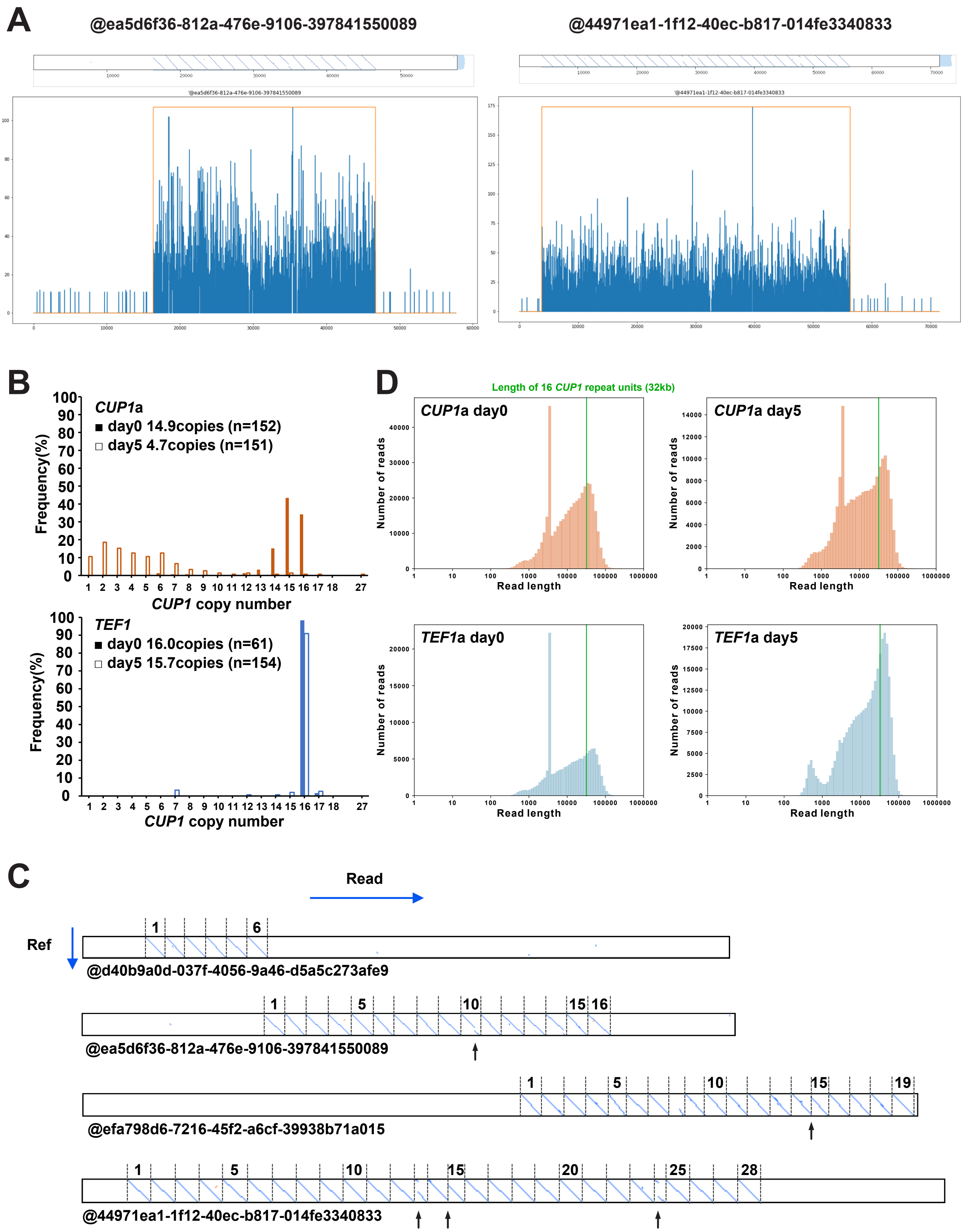

Doi et al., Supplementary Figure S3

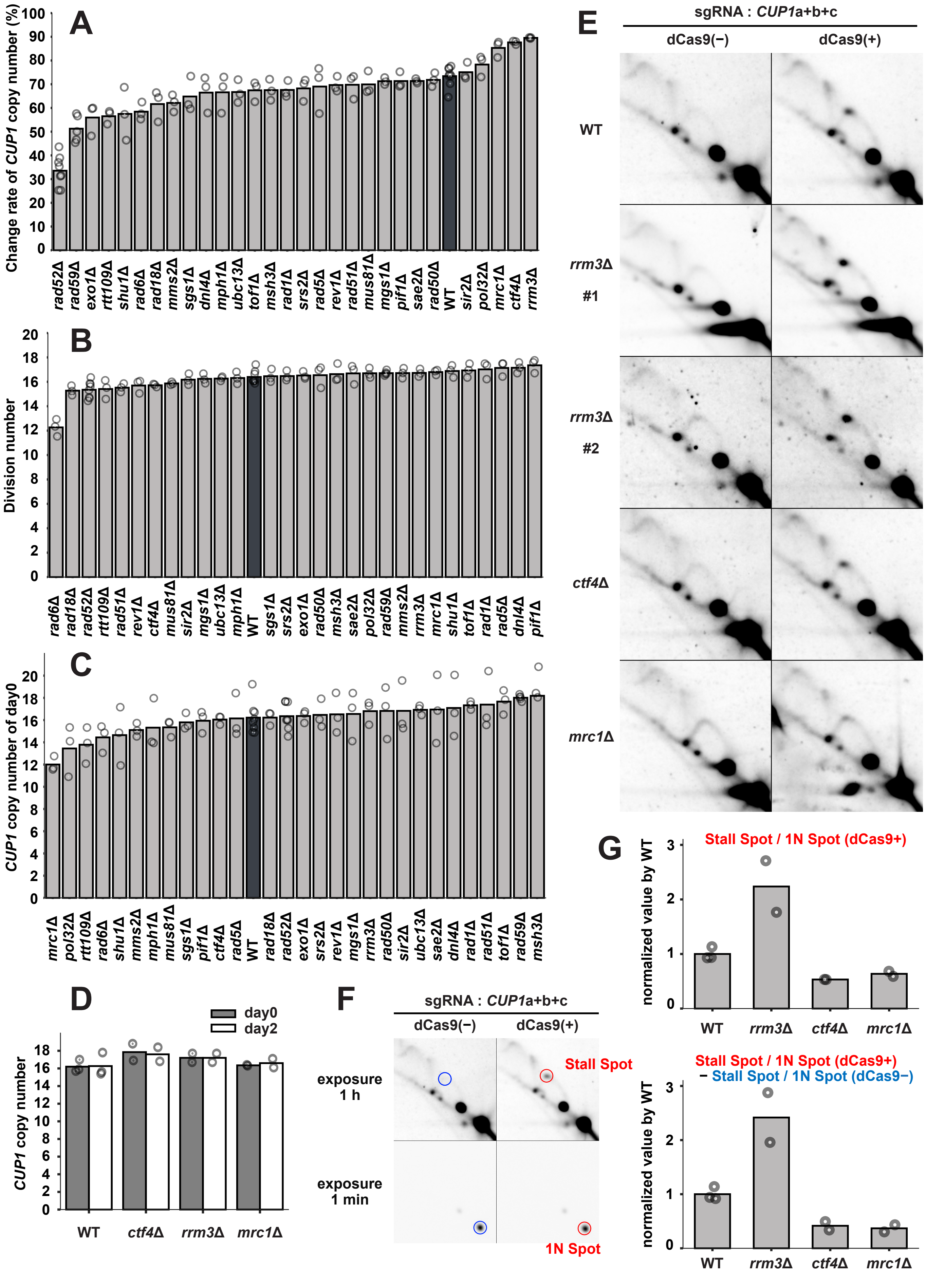

Doi et al., Supplementary Figure S4

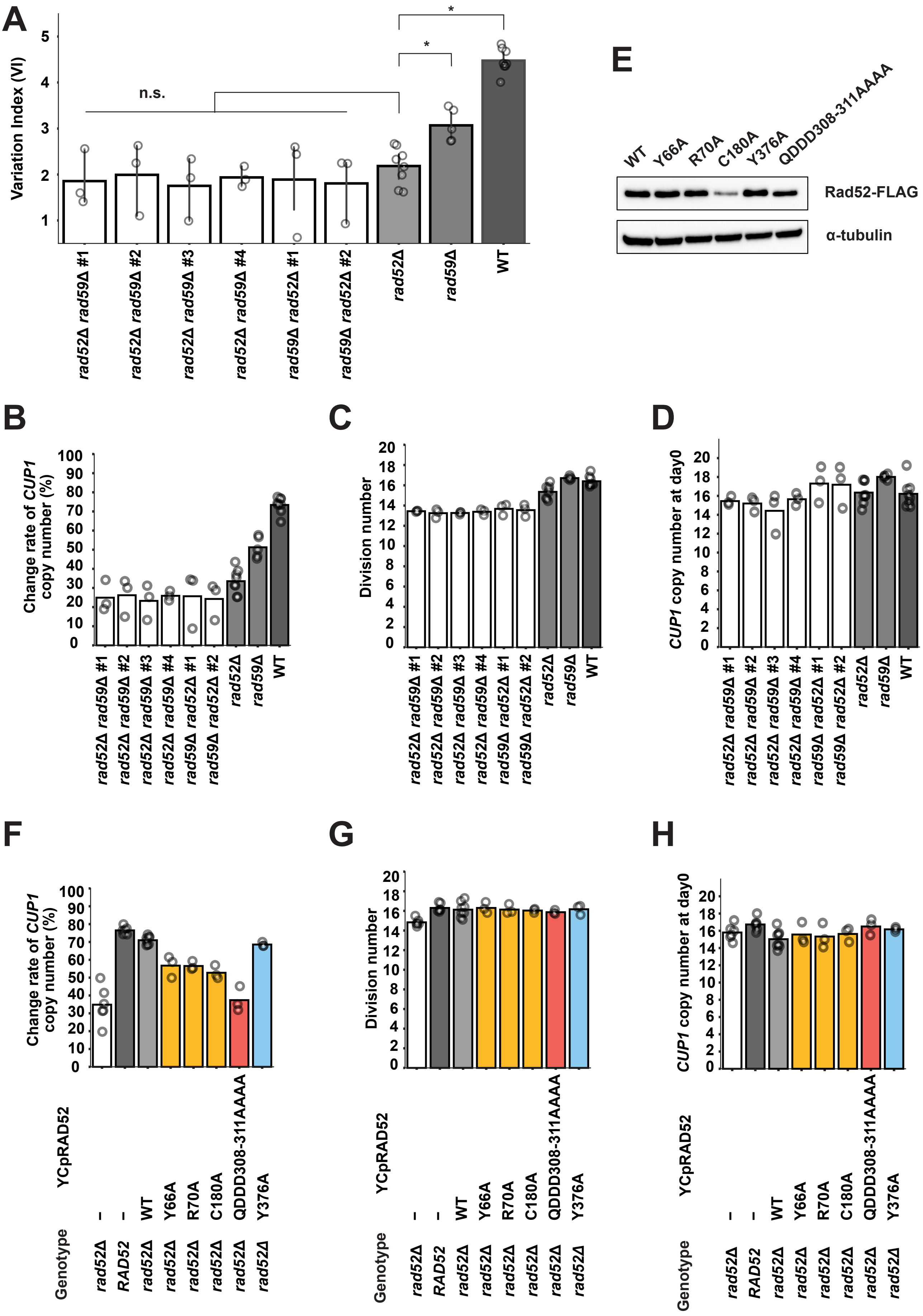

Doi et al., Supplementary Figure S5
